# 

**Published:** 1878-01

**Authors:** 


### BOOKS AND PAMPHLETS RECEIVED.

Transactions of the New York Pathological Society. Vol. II. Based on the proceedings of the year 1875 and, largely supplemented from the records of 1844–1877. Edited by John C. Peters, M. D., President Medical Society County of New York. New York: William Wood & Co. 1877. 8vo.; cloth.

Transactions of the International Medical Congress of Philadelphia, 1876. Edited for the Congress by John Ashleman, Jr., A. M., M. D. Philadelphia: Printed for the Congress. 1877. 8vo.; cloth, pp. 1153.

Transactions of the Medical Society of the State of Pennsylvania at its Twenty-eighth Annual Session, June 13, 1877. Vol. XI. Part 2. Philadelphia: Collins & Co. 1877. Paper, 8vo.; pp. 830.

Materia Medica for the Use of Students. By John J. Biddle, M. D., etc. Eighth Edition. Revised and enlarged, with numerous illustrations. Philadelphia: Lindsay & Blakiston. 1878. 8vo.; cloth, pp. 462.

A Compend of Diagnosis in Pathological Anatomy, with Directions for Making Post-Mortem Examinations. By Johannes Orth. Translated by F. S. Shattuck, M. D., and George Kraus Sabine, M. D. Revised by Reginald Heber Fity, M. D., with Numerous Additions from MS., Prepared by the Author. Sole authorized English edition. New York: Hurd & Houghton. Boston: H. O. Houghton & Co. Cambridge: The Riverside Press. 1878. 8vo.; cloth.

Modern Medical Therapeutics; a Compendium of Recent Formulæ and Specific Therapeutical Directions from the Practice of Eminent Contemporary Physicians, American and Foreign. By George H. Napheys, A. M., M. D., etc. Fifth edition, enlarged and revised. Philadelphia: D. G. Brinton. 1878. 8vo. cloth; pp. 600. Cloth, $4; full leather, $5.

The Action of Medicine. By Isaac Ott, A. M., M. D., etc., etc. With twenty-two illustrations. Philadelphia: Lindsay & Blackiston. 1878. 8vo.; cloth, pp. 168.

The Physician's Hand-Book for 1878. By William Elmer, M.D., and Albert D. Elmer, M.D. New York: W. A. Townsend. 1878.

Diseases of the Nasal Cavity and Pharynx. Translation from the German of Dr. Carl Michel, of Cologne on the Rhine. With an introduction by E. L. Shurlyan. C. C. Yeaman. First American edition. Detroit. 1877: 8vo.; paper, pp. 109.

Fourth Biennial Report of the State Board of Health of California, for the years 1876 and 1877. Sacramento State Office: 8vo.; cloth, pp. 92.

Lecture 1: Clinical Lectures on Surgery. By J. H. Pooley, M.D. (Reprint from the *Ohio Medical and Surgical Journal.*)

Pyæmia and Septicæmia. By B. A. Watson, M.D. Reprint, *New York Medical Journal*, October and November, 1877.

Report of Cæsarean Section after Seven Days' Labor, with some Comments upon the Operation. By Edward W. Jenks, M.D. Reprint from *The American Journal of Obstetrics and Diseases of Women and Children.* Vol. X., No. IV.; October, 1877.

Mechanism of Joints. By Harrison Allen, M.D. Extracted from the Trans. of the International Medical Congress. Philadelphia: September, 1876.

The Localization of Diseased Action in the Œsophagus. By Harrison Allen, M.D. Reprint from the *Phila. Med. Times.*

Note on the Anatomy of the Perineum. By Harrison Allen, M.D. Extracted from the Trans. College of Physicians of Philadelphia. Third series, vol. II.

Third Annual Report of the Executive Committee of the Asylum at Walnut Hill, Hartford, Conn., at their Annual Meeting, Oct. 8, 1877. Also petition to the Legislature.

Condensed Statement of Mortality in the City of Chicago, for the weeks ending, Oct. 13, Nov. 3, Nov. 10, Nov. 17. O. C. De Wolf, Commissioner of Health; H. P. Wright, Registrar of Vital Statistics.

### ANNOUNCEMENTS FOR THE MONTH.

### SOCIETY MEETINGS.

Chicago Medical Society—Mondays, Jan. 7 and 21.

Chicago Society of Physicians and Surgeons—Mondays, Jan. 14 and 28.

### CLINICS.

MONDAY.

Eye and Ear Infirmary—2 to 4 p. m., by Prof. Holmes and Dr. Hotz.

Mercy Hospital—2 to 3 p. m. Surgical, by Prof. Andrews.

Rush Medical College—1:30 p. m. Medical, by Dr. Bridge.

County Hospital—8 p. m. Necropsy, by Dr. Danforth.

Woman's Medical College—3 p. m. Surgical, by Prof. Owens.

TUESDAY.

County Hospital—1:30 p. m. Medical, by Prof. Bevan; 2:30 p. m. Surgical, by Dr. Bogue.

Mercy Hospital—2 p. m. Medical, by Prof. Hollister.

Eye and Ear Infirmary—2 p. m. Prof. Jones.

WEDNESDAY.

County Hospital—1:30 p. m. Gynecological, by Prof. Fitch; 2:30 p. m. Ophthalmological, by Dr. Montgomery.

Mercy Hospital—2 p. m. Eye and Ear, by Prof. Jones.

Rush Medical College—4 p. m. Diseases of the Chest, by Prof. Ross.

THURSDAY.

Mercy Hospital—2 p. m. Medical, by Prof. Davis.

Rush Medical College—1:30 p. m. Neurological, by Prof. Lyman.

Eye and Ear Infirmary—2 to 4 p. m. Operations by Prof. Holmes and Dr. Hotz.

FRIDAY.

Mercy Hospital—2 p. m. Medical, by Prof. Davis.

County Hospital—1:30 p. m. Medical, by Prof. Ross; 2:30 p. m., Surgical, by Prof. Gunn.

Woman's Medical College—10 p. m. Ophthalmological, by Dr. Montgomery.

SATURDAY.

Rush Medical College—2 p. m. Surgical, Prof. Gunn.

Chicago Medical College—2 p. m. Surgical, by Prof. Andrews and Isham; 3 p. m., Diseases of the Chest, by Prof. Johnson.

Woman's Medical College—12 m. Gynecological, by Prof. Fitch; 3 p. m. Dermatological, Dr. Maynard.

Special Clinics daily, from 2 to 4 p. m., at the South Side Dispensary, and at the Central Free Dispensary.

For schedule of lectures at the colleges, apply to the college janitors.

